# Improvement of Cassava Varieties for High Nutritional Quality Adapted to the Pacific and Andean Regions in Colombia

**DOI:** 10.3390/plants14243762

**Published:** 2025-12-10

**Authors:** Eberto Rodríguez, Amparo Rosero, José Ives Perez, Lina Garavito, Juan Carlos González, Karen Alarcón, Nelson Morante, Sandra Salazar, John Belalcazar, Hernán Ceballos

**Affiliations:** 1 Centro de Investigación Palmira, Corporación Colombiana de Investigación Agropecuaria—AGROSAVIA, Palmira 763533, Colombia; 2 Centro de Investigación Obonuco, Corporación Colombiana de Investigación Agropecuaria—AGROSAVIA, Pasto 520038, Colombia; 3 Centro de Investigación Palmira-Sede Popayán, Corporación Colombiana de Investigación Agropecuaria—AGROSAVIA, Popayán 190003, Colombia; 4 Centro de Investigación El Mira, Corporación Colombiana de Investigación Agropecuaria—AGROSAVIA, Tumaco 528517, Colombia; 5 Alliance of Bioversity International and CIAT, The Americas HUB, Km 17 Recta Cali-Palmira, Palmira 763537, Colombia

**Keywords:** cyanogenic potential, HCN, β-carotene, pro-vitamin A carotenoids, biofortification, cooking time

## Abstract

Increased levels of pro-vitamin A carotenoids in cassava (*Manihot esculenta* Crantz) roots is a valuable contribution toward reducing widely spread vitamin A deficiency in vulnerable human populations worldwide. This study aimed to evaluate five yellow-fleshed cassava genotypes with higher β-carotene contents for fresh consumption in the Cauca River Valley and the Pacific regions of Colombia. Agronomic performance, productivity, and culinary quality were assessed across four locations. The results showed that two yellow-fleshed genotypes had adequate performance in the subregions. *SM3677-74* was identified for the Cauca River Valley subregion, and *GM3650-51* for the Pacific subregion. These genotypes showed competitive performance compared to the regional checks (often outperforming them) and showed good adaptability to the target environments. The excellent productivity and enhanced nutritional quality (>5 µg/g β-carotene and >11 µg/g total carotenes) of these genotypes make them suitable for potential for release as new varieties in those specific subregions. The experimental genotypes demonstrated acceptable quality for consumption, with low HCN content (less than 50 µg/g) and cooking time was <30 min. The successful adaptation and superiority of improved cassava genotypes ensure the future availability of carotenes-enhanced cassava varieties the Pacific and Andean Regions in Colombia.

## 1. Introduction

Cassava (*Manihot esculenta* Crantz) is a highly efficient crop for starch accumulation under conditions of limited water and nutrient availability, particularly when compared with other major starchy crops such as maize, wheat, and rice [1,2]. In 2023, global cassava production was approximately 330 million metric tons, with Africa contributing about 204 million tons, followed by Asia with 89 million tons and the Americas with 28 million tons [3]. In Colombia, the area planted with cassava for fresh consumption in 2022 in the departments of Cauca, Nariño, and Valle del Cauca totaled 6720 hectares, yielding 95,903 tons of fresh roots, with an average productivity of 14.3 t ha^−1^ [4]. In these departments, cassava production is primarily aimed at ensuring food security, with surplus harvests entering local markets. Cassava serves as a vital source of carbohydrates, particularly in rural communities where other crops may fail due to poor soils or adverse climatic conditions [5,6]. Although rich in energy, cassava is relatively low in protein and micronutrients [7]. However, yellow-fleshed varieties have been biofortified to provide significant levels of β-carotene, a precursor of vitamin A [8,9]. From a digestive standpoint, cassava is generally considered to have low allergenic potential, although its high glycemic index may pose risks for individuals with diabetes if consumed in large quantities or without complementary sources of fiber or protein [7].

The breeding process must ensure a continuous supply of new improved germplasm adapted to the prevailing climatic conditions of each region, thereby minimizing the impact of biotic and abiotic constraints on production systems [10]. Among the major achievements of cassava breeding are the development of high-yielding varieties with resistance to key diseases and insects, as well as tolerance to abiotic stresses including drought and low soil fertility [11]. Recent advances have accelerated the genetic improvement process; flower-induction techniques have significantly shortened breeding cycles by promoting earlier flowering, reducing the time from 6 to 7 months to 3 to 4 months in late-flowering genotypes [12]. The use of biotechnological tools, including marker-assisted selection, genomic selection, and gene editing through CRISPR-Cas9, may facilitate the development of genotypes with increased disease resistance, improved nutritional quality and starch content, and enhanced drought tolerance [13,14].

Enhancing the nutritional value of food adds value to agricultural products, strengthens food security, and helps address the global dietary dependence on a limited number of staple crops that are energy-dense but nutritionally poor, a trend that negatively affects human health [15]. Biofortified cassava varieties with elevated levels of provitamin A carotenoids have been developed and released in sub-Saharan Africa and Latin America by national breeding programs and initiatives such as HarvestPlus [9,16,17]. These varieties aim to combat vitamin A deficiency, a major public health concern associated with impaired vision, immune dysfunction, and increased child mortality [8]. The integration of nutrient-dense cassava into local food systems therefore represents a promising strategy to improve dietary diversity and reduce hidden hunger in food-insecure regions [18].

In Colombia, stunted growth and chronic malnutrition are most prevalent among children of less-educated mothers living in rural areas, particularly in the Atlantic, Amazon, Orinoquía, and Pacific regions [19] Micronutrient deficiencies, especially in iron and vitamin A, remain a significant public health concern [20]. Young children are the most vulnerable group, with 28% of those aged 12–23 months and one in four children aged 1–4 years suffering from vitamin A deficiency. Breeding efforts have focused on generating high-yielding genotypes with enhanced root and dry matter productivity to meet the specific demands of both agro-industrial processing and fresh consumption [21]. More recently, the need to develop biofortified genotypes has been recognized as a strategy to address food security challenges, extreme poverty, and public health concerns.

In the Cauca River Valley (CRV) and Pacific subregions of Colombia, Agrosavia, CIAT, and HarvestPlus (Palmira, Colombia) supported the identification of yellow-fleshed genotypes (β-carotene content > 4 μg/g) for agronomic evaluation. The objective of this study was to identify at least one high-β-carotene variety to enhance varietal options and contribute to reducing chronic malnutrition and vitamin A deficiency in these regions of Colombia.

## 2. Material and Methods

### 2.1. Plant Material

The Agronomic Evaluation Trial (AET) was officially registered in 2019. The cassava genotypes used in this evaluation were selected from five years of agronomic performance trials (2016–2020) conducted in the CRV and Pacific subregions (Table 1). These trials identified promising genotypes to be registered as improved varieties in the National Registry of Commercial Cultivars of the Colombian Agricultural Institute (ICA) (Bogotá, Colombia), based on their adaptability to each subregion.

In Colombia’s inter-Andean valleys, the only cassava cultivar officially registered in the National Registry of Commercial Cultivars of the Colombian Agricultural Institute (ICA) is *Manihotica P-13*, released in 1986 [21,22]. As the sole registered variety for this agroecological zone, *P-13* serves as the commercial check for the Andean subregion. In contrast, the Pacific subregion currently lacks ICA-registered cassava cultivars for commercial distribution. Therefore, two locally adopted landraces (*Eco-Blanca* and *Llanera*) were used as regional checks. These regional landraces are favored by local producers for their palatability, good performance, and high productivity, but they have not been formally registered due to lack of evaluation and unknown genetic origin. The Llanera variety, associated with cultivation in the Orinoquia region, has been observed under Pacific Coast growing conditions [23].

Since there are no yellow-fleshed cassava varieties with high β-carotene content in the National Registry of Commercial Cultivars (ICA), white-fleshed genotypes were used as controls to assess relevant agronomic attributes such as fresh root yield (FRY) and dry matter content (DMC) in the roots.

Crosses among selected progenitors were made at CIAT’s Experimental Station in Palmira, Valle del Cauca, to produce recombinant sexual seeds. The conventional breeding method for cassava relies on phenotypic recurrent selection and requires several years of field evaluation [21]. Heritability of carotenoid content in cassava roots, the primary trait for selection, is high [24]. Therefore, a modification on the breeding scheme was introduced by shortening the duration of each cycle of selection [25].

### 2.2. Locations

The municipalities selected within CRV and Pacific subregions in Colombia share a certain degree of agro-environmental uniformity. However, statistically significant differences may still occur in terms of environmental favorability, which would primarily reflect variations in soil characteristics (Table 2).

### 2.3. Experimental Design and Management

A Randomized Complete Block Design (RCBD) was used as experimental design. Three and four replications per location were used in the CRV subregion and the Pacific subregion, respectively. The planting density was 10,000 plants/ha, with 1 m spacing between rows and 1 m between plants. The experimental plots consisted of five rows, each containing five plants, for a total of 25 plants. Data was obtained from the nine central plants, with border plants excluded from the evaluations.

Soil preparation and planting were carried out according to the edaphic and agroclimatic conditions of each area. Weed management included the application of pre-emergent herbicides, graminicides, and herbicide screens to prevent phytotoxicity to the crop. Fertilization was performed according to the results of soil analyses, and in accordance with the crop requirements, which specify that for every ton of roots produced, 4.42 kg/ha of nitrogen, 0.67 kg/ha of phosphorus, 3.58 kg/ha of potassium, 1.36 kg/ha of calcium, and 0.82 kg/ha of magnesium are absorbed. The genotypes were harvested 10 months after planting (MAP). Regarding pests and diseases, no particular control practices were implemented.

### 2.4. Agronomic Evaluation

Plant type is an integral trait related to architecture, height of first branching, and vigor, and was evaluated through visual observation of all evaluated genotypes using the following scale: (1) clearly better than average, (2) slightly better than average, (3) average, (4) slightly worse than average, and (5) clearly worse than average. A similar scale was used for vigor and root type. Qualitative variables of root quality were observed to determine shapes, sizes, bark and root-flesh color, root shape, and presence of root constrictions. Colors suitable for commercial production total and commercial FRY were calculated based on parameters collected from each plot (commercial and total root weight). DMC was determined using the gravimetric method [26].

### 2.5. Evaluation of Nutritional Quality of Roots

Upon harvest, roots were shipped to Palmira. At CIAT’s root quality laboratory, key nutritional traits were assessed: cyanogenic potential (HCN) was evaluated using forty grams of grated pulp from fresh roots, which were homogenized in extracting solution. The resulting mixture was centrifuged, filtered, and mixed with the enzyme linamarase. Additional procedures were performed as previously described [27]. Total carotenoids (TCCs) and β-carotene (TBC) were predicted on uncooked fresh roots through a well-established protocol using near-infrared spectroscopy [25].

### 2.6. Cooking Quality and Sensory Test

Cooking quality of boiled roots (texture, bitterness, external appearance, color, etc.) from different genotypes was assessed sensorially using a hedonic scale. The scale consisted of five categories, ranging from “extremely dislike” to “extremely like.” This scale was applied to evaluate preferences based on a 5-point hedonic scale.

High-quality culinary cassava roots have an optimal cooking time of 20–30 min. With regard to root selection and harvesting from representative and healthy plants with special efforts to prevent root damage, only commercial roots were used, and the procedure for sampling was used according to previous studies [17]. Culinary quality of the cooked pieces was evaluated by a hedonic test which considered texture (hard/glassy, soft, and firm), flavor (bitter, neutral, and sweet), and fiber content (acceptable and non-acceptable). The scale had five categories, ranging from “extremely dislike” to “extremely like”. All genotypes were renamed with randomized codes and specific conditions, as detailed in previous studies [17].

### 2.7. Statistical Analysis

Data obtained from the field were digitized into databases using the Microsoft Excel^®^ program for review and curation. These processes included descriptive statistics to identify outliers or possible typing errors. The collected data were analyzed based on a multilocational experiment, as described by Martínez et al. [28] and Littell et al. [29]. A mixed-model analysis of variance (ANOVA) was employed, considering the fixed effects of genotype, location, and the interaction between these factors. The random effects considered were the locations within each region and the blocks within each location. The following equation describes the statistical model used:

$$y_{ijkn}=\;\mu +\;G_{i}+\;R_{j}+\;L_{n}+B_{k(jn)}+{GR}_{ij}+\;e_{ijkn}$$

$y_{ijkn}$ = Measured random variable;

μ = Overall average;

*G_i_* = Effect of *i*-th genotype;

*R_j_* = Effect of *j*-th region;

*L_n_* = Effect of *n*-th location $idd\;N(0,\sigma_{l}^{2})$;

*B_k_*_(*jn*)_ = Effect of *k*-th block $idd\;N(0,\sigma_{b}^{2})$;

*GR_ij_* = Effect of interaction between the *i*-th genotype and the *j*-th region;

*E_ijkn_* = Experimental error $idd\;N(0,\;\sigma^{2})$.

Prior to conducting the analysis of variance (ANOVA), tests for normality and homogeneity of variances were performed. The data were then analyzed using generalized ANOVA, employing an appropriate residual distribution based on the nature of the variables, such as Poisson, Beta, Gamma, among others. The selection of the appropriate residual distribution was based on a likelihood ratio of models and the analysis of model selection criteria, such as the Akaike Information Criterion (AIC) and the Bayesian Information Criterion (BIC).

In cases where statistical differences were found, post-ANOVA analysis was carried out using Tukey’s method for the sources of variation in genotype and environment, as well as for cases where the G × A interaction showed significant statistical differences. Additionally, a planned comparison test of mean values between genotypes for the response variables was performed, even if the ANOVA did not show statistical differences a priori, using the Dunnett statistic. A significant level (α) of 0.05 was considered for all analyses.

For qualitative variables related to growth and root characteristics, the most frequent value for each trait was determined using the Microsoft Excel^®^ database. Statistical analysis was conducted using SAS/STAT software V 9.4.

## 3. Results

### 3.1. Agronomic Performance

The analysis of variance revealed significant differences (*p* ≤ 0.01) among genotypes for FRY but not for DMC. Dry root yield (DRY, t ha^−1^), which combines these two variables, also showed significant differences among genotypes (Table 3). The effects of location and the location-by-genotype interaction were significant for all three variables (FRY, DMC, and DRY).

**Figure 1:**
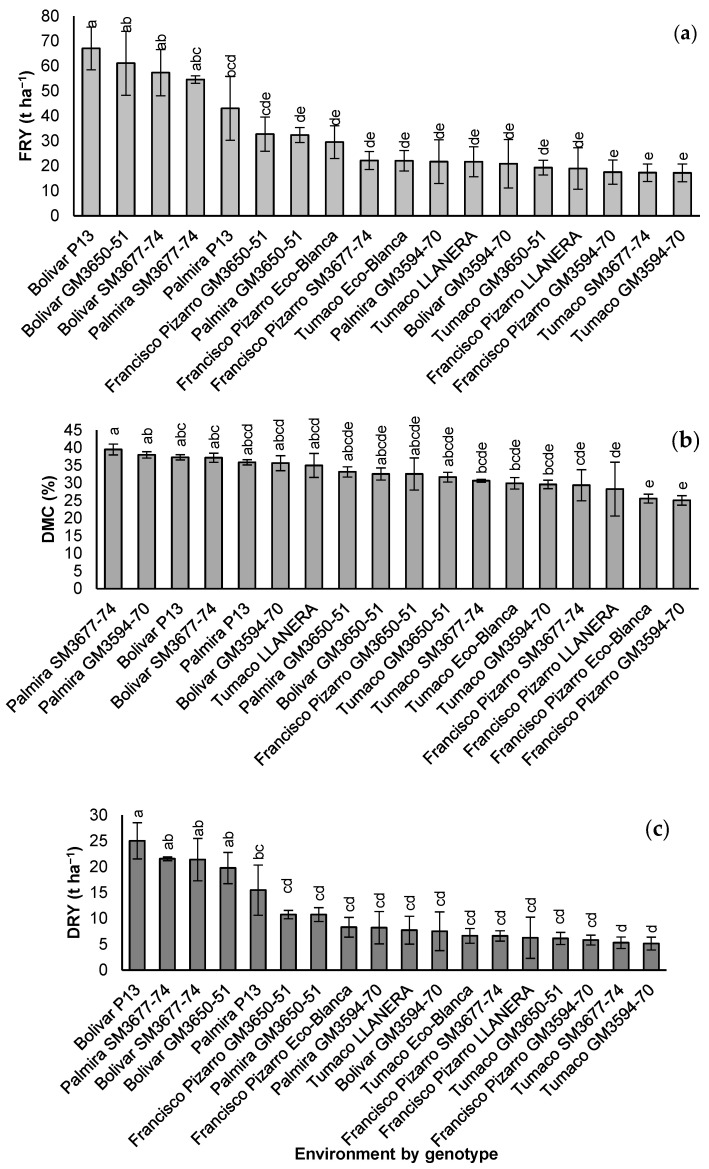
Comparison of mean values for (**a**) fresh root yield (FRY); (**b**) dry matter content (DMC); and (**c**) dry root yield (DRY) among six cassava genotypes evaluated in the Pacific and CRV subregions. Mean values with the same letter are not significantly different at *p* ≤ 0.05 (Tukey’s test).

The highest average FRY values were recorded in the CRV subregion at Bolívar (51.6 t ha^−1^) and Palmira (37.9 t ha^−1^), while the Pacific subregion locations, Francisco Pizarro and Tumaco, showed considerably lower averages (24.2 and 19.5 t ha^−1^, respectively). The Pacific subregion is characterized by frequent and often excessive rainfall, which reduces cassava productivity [30]. FRY of individual genotypes within a single location ranged from 67 t ha^−1^ for *P13* at Bolívar to 17 t ha^−1^ for *GM3594-70* at Tumaco (Figure 1a). At Bolívar, the FRY of two experimental clones—*GM3650-51* (61 t ha^−1^) and *SM3677-74* (57 t ha^−1^)—were not statistically different from that of *P13*, which served as the check for this location. Similarly, at Palmira, *SM3677-74* (55 t ha^−1^) showed greater (although not significantly) *P13* (43 t ha^−1^) and *GM3650-51* (32 t ha^−1^). In the Pacific subregion, the highest FRY was observed for *GM3650-51* (32 t ha^−1^) at Francisco Pizarro, but this value did not differ significantly from those of the two regional checks, *Eco-Blanca* and *Llanera*. In contrast, *GM3594-70* showed consistently poor FRY performance across all locations.

DMC averages varied significantly across locations (Table 3), and genotype-by-location interactions were also significant. However, differences among genotypes did not reach statistical significance. Similar to FRY, locations in the CRV subregion (Bolívar and Palmira) tended to show the highest DMC values, averaging 36.1%, compared with 31.7% in the Pacific subregion. The highest individual averages within a single location were recorded for *SM3677-74* and *GM3594-70* at Palmira, followed by *P13* and *SM3677-74* at Bolívar (Figure 1b). Thus, genotype *SM3677-74* exhibited excellent DMC levels in the more productive CRV locations. Illustrating the importance of G × E interaction, *GM3650-51* performed modestly in the CRV but achieved notably higher DMC averages in the Pacific subregion (33% at Francisco Pizarro and 32% at Tumaco).

Overall productivity—reflected by dry root yield (DRY), which combines FRY and DMC—is closely linked to starch production per unit area. Genotype, location, and their interaction all had significant effects on DRY (Table 3). As expected, the highest DRY averages were observed in the CRV locations (Bolívar and Palmira; Figure 1c). *P13* at Bolívar (25 t ha^−1^), *SM3677-74* at Palmira and Bolívar (22 and 21 t ha^−1^, respectively), and *GM3650-51* at Bolívar (20 t ha^−1^) exhibited significantly higher DRY values than the other genotypes within the same or different locations. *P13* at Palmira showed an intermediate average (15 t ha^−1^), while the remaining genotypes averaged around 10 t ha^−1^ or less. *GM3650-51* also achieved the highest DRY in the Pacific subregion at Francisco Pizarro (around 11 t ha^−1^), comparable to its performance at Palmira, whereas its yield at Tumaco was average.

### 3.2. Visual Descriptors of the Plant and the Roots

**Figure 2:**
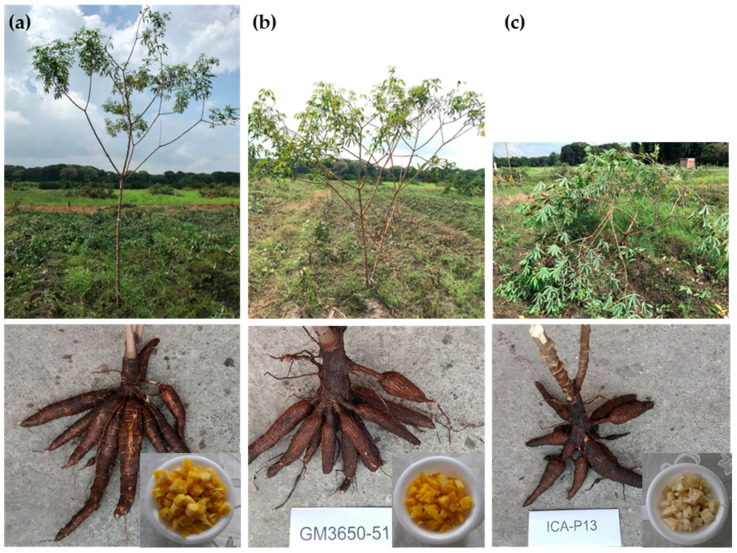
Visual illustration of plant type, roots, and boiled cassava appearance of promising genotypes. (**a**) *SM3677-74*, (**b**) *GM3650-51*, (**c**) *P13*.

Plant development was excellent, with vigor ranging from intermediate in *P13* to vigorous in the other genotypes (Figure 2, Table 4). No lodging was observed for any genotype or location. The plant type scores of *SM3677-74* and *GM3650-51* indicated a clearly superior architecture compared with the average, while *GM3594-70* was slightly above average.

Visual inspection of the roots confirmed the presence of only a few constrictions, a desirable trait. Skin color ranged from light to dark brown. As expected, the flesh color of the experimental clones was yellow, while that of the checks was white. In no case was the peduncle or “neck” length excessive. Roots from *P13* did not have peduncle, which is an undesirable feature. Root type scores for the experimental clones were consistently average or above average, with *GM3650-51* showing a clearly superior score in the Pacific subregion.

### 3.3. Nutritional Quality of Roots

Table 5 summarizes the results for key nutritional traits of cassava roots. HCN was consistently below 50 µg/g, well under the maximum allowable threshold [31]. *GM3594-70* and *SM3677-74* exhibited the highest TBC and TCC, particularly in the CRV subregion. In contrast, *GM3650-51* showed higher carotenoid levels in the Pacific subregion. As expected, the white-fleshed genotypes displayed considerably lower β-carotene and total carotenoid contents.

### 3.4. Cooking Quality and Consumer Acceptability

Overall, the assessments of color, taste, and texture after boiling were acceptable. None of the genotypes were considered unacceptable, although none were particularly preferred (Table 6). Cooking times for the three experimental clones were within acceptable limits [21,32] but tended to approach the upper threshold, particularly in the Pacific subregion.

## 4. Discussion

Plant development was excellent, with vigor ranging from intermediate to vigorous. The evaluation process preceding the final AET stage proved effective in selecting genotypes with desirable plant architecture. In the field, excessively high plants are undesirable due to increased vulnerability to lodging, which, in turn, promotes early sprouting in pre-harvested plants [26]. Therefore, adequate architecture is not only related to better conditions for crop management but also defines the planting material for the next crop cycle, a problem that is observed in variety P13.

There was no significant genetic variation for DMC. In recent years, it has become evident that increasing carotenoid concentration in cassava roots often incurs a penalty in terms of DMC [17,33]. In this study, however, the negative correlation between DMC and TBC/TCC was not evident. This may be partly due to the limited number of genotypes evaluated and the rather moderate DMC levels observed in the white-fleshed checks, particularly Eco-Blanca. The highest DMC values are typically found in varieties bred for industrial uses such as starch, animal feed, or bioethanol production, where high DMC is a key requirement. None of the checks used here were selected for industrial processing but rather for their cooking quality, which does not demand high DMC. Furthermore, environmental conditions in the two Pacific subregion locations (Tumaco and Francisco Pizarro) were not conducive to high DMC levels.

DMC showed significant variation among locations. The average DMC in the CRV subregion was considerably higher than in the Pacific subregion (36.14% vs. 31.66%). *SM3677-74* exhibited excellent average DMC levels across the two CRV locations (38.4%) but only average values in the Pacific subregion (29.99%). In contrast, *GM3650-51* showed above-average DMC in the Pacific locations (32.1%). DMC, together with plant type score, are valuable traits for identifying germplasm particularly adapted to specific environments [34].

Location and genotype-by-environment interaction effects were significant for all three agronomic traits (FRY, DMC, and DRY). Substantial environmental differences between the CRV and Pacific subregions clearly influenced productivity and quality, suggesting adaptation of the genotypes to specific environmental conditions. Under these findings, several studies reported significant interactions between the environment and cassava cultivars, representing an opportunity to identify the best discriminating environments and select stable genotypes in different environmental conditions and, alternatively, it may be desirable to select clones adapted to specific environments [26]. Thus, according to results found in this study, the genotype *SM3677-74* demonstrated excellent adaptation to the CRV subregion but performed poorly in the two Pacific subregion locations, contributing to the significant G × E interaction observed for the three variables listed in Table 3. This genotype, however, displayed an outstanding plant type score in both subregions. *GM3650-51* showed acceptable performance in the CRV subregion and outstanding performance at the Francisco Pizarro location. Its plant type score ranged from above average to outstanding across both subregions. This behavior suggested that specific selection for each environmental region, strong environmental effects, and significant genotype-by-environment interactions pose a challenge for cassava breeders and highlight the importance of conducting evaluations across multiple locations. AMMI/GGE and multi-attribute models can be particularly useful for identifying genotypes with stable performance and superior adaptation to the target environments [35,36,37].

The root systems of the three experimental genotypes did not present any particular issues and were within acceptable thresholds. They exhibited only minor constrictions, and their peduncles or “necks” were of adequate length, neither excessively long nor absent. More importantly, their HCN levels were well within acceptable limits, and the roots were not bitter. The color, taste, and texture of boiled roots from the experimental clones were also within acceptable ranges, although cooking time was close to the upper threshold. Interestingly, HCN levels tended to be higher in the Pacific subregion, which contrasts with reports indicating that limited water availability (drought) increases HCN accumulation in roots [1,38,39].

*GM3594-70* and *SM3677-74* exhibited the highest TBC and TCC, particularly in the CRV subregion. In contrast, *GM3650-51* showed higher carotenoid levels in the Pacific subregion. These results showed the influence of environment to carotenes accumulation, which is consistent with previous studies [40,41], being another suggestion to proceed with specific varietal selection for determined environmental conditions. As expected, the white-fleshed genotypes displayed considerably lower β-carotene and total carotenoid contents.

Color, taste, and texture are critical traits determining consumer acceptance of boiled cassava roots [42]. Hedonic assessment of cooking quality was carried out by an ad hoc panel composed of untrained farmers, students, and household women. Notably, there were marked cultural and ethnic differences among participants from the various locations. As sensory perception is inherently subjective, evaluations conducted by untrained panelists represent valuable information; however, they can be limited by personal perception [43]. This study has proven root crops to be highly correlated with quality factors, since texture, flavor, and fiber can be felt by panelists and the decision is related to those factors measured by laboratory analysis [17,44].

## 5. Conclusions

Genotype *GM3650-51* exhibits valuable agronomic and commercial traits for cultivation in the Pacific subregion as a yellow-fleshed cassava variety intended for fresh consumption. This genotype achieved a high FRY (26 t ha^−1^), together with acceptable DMC (32.1%) and DRY (8.44 t ha^−1^), thereby enhancing the economic and nutritional value of cassava in this subregion. Its distinguishing feature is its β-carotene concentration of 5.1 ppm, which offers a nutritional advantage not previously available in cassava cultivars grown in the region.

Currently, no improved cassava cultivars from the Pacific subregion of Colombia are registered in the National Registry of Commercial Cultivars (ICA). The traits of *GM3650-51*—together with other desirable agronomic characteristics such as plant vigor, height to first branching, sprouting capacity, and root quality—constitute a valuable contribution to the regional cassava production sector.

Genotype *SM3677-74* also exhibited outstanding attributes as a potential new commercial cultivar for fresh consumption in the CRV subregion. It combines a high FRY, comparable to that of the commercial check *P13*, with the added nutritional advantage of elevated β-carotene content. In addition, this genotype displays excellent plant architecture and root morphology, further reinforcing its potential for registration as a commercial variety for the target environment.

## Figures and Tables

**Table 1:** Characteristics of the genotypes established in agronomic evaluation trials in representative areas of the CRV and Pacific subregions.

Genotype	Flesh-Root Color	Genealogy (♀ × ♂)
*SM3677-74*	Yellow	*GM905-60* × father unknown
*GM3650-51*	Yellow	*GM1561-11* × *GM1551-20*
*GM3594-70*	Yellow	*GM1561-11* × *GM1548-33*
*P13* *	White	Botanical seed irradiated with gamma rays. Open pollination of female progenitor *CMC-9*
*Eco-Blanca* **	White	Landrace of unknown pedigree
*Llanera* **	White	Landrace of unknown pedigree

(*) Commercial check for the Cauca River Valley subregion; (**) regional check for the Pacific subregion.

**Table 2:** Locations where agronomic evaluation trials (AET) were conducted during the years 2020–2021.

Subregion	Department	Municipality	Additional Information
Río Cauca Valley	Valle del Cauca	Palmira	AET at Palmira. Experimental plot Agrosavia—C.I. Palmira. 3°30′46.0″ N; 76°18′51.2″ W.
		Bolivar	AET at Bolivar. Vereda Ricaurte. 4°18′27.0″ N 76°11′47.0″ W.
Pacific	Nariño	Tumaco	AETat Tumaco. Experimental plot Agrosavia—C.I. El Mira. 01°32′56.4″ N; 078°41′56.0″ W.
		Francisco Pizarro	AET at Francisco Pizarro. Vereda Salahonda. 02°1′53.13″ N; 78°39′19.48″ W.

**Table 3:** Mean squares from the analysis of variance for yield-related variables in cassava genotypes with high β-carotene content evaluated in four locations across two subregions.

Source of Variation	df	Fresh Root Yield (t ha^−1^)	Dry Matter Content (%)	Dry Root Yield (t ha^−1^)
Genotype	5	643.42 **	19.03 ns	72.63 **
Location	3	1590.27 **	166.57 **	226.66 **
Rep/Location	10	67.16 ns	8.76 ns	7.04 ns
Genotype × Location	9	305.62 **	26.67 **	46.93 **
Error	28	43.28	8.63	6.32
Coefficient of variation		21.16	9.13	23.40

df: degrees of freedom; CV: Coefficient of variation; ** Significant at *p*: 0.01; ns: Non-significant.

**Table 4:** Qualitative variables related to root and plant characteristics of six cassava genotypes under agronomic evaluation for fresh consumption with high β-carotene content in four locations across two subregions.

Genotype	Root Traits							Plant Traits		
	Type	Root Length	Neck Length	Skin Color	Flesh Color	Shape	Constrictions	Lodging	Type	Vigor
*Pacific Subregion*										
*SM3677-74*	2	S	I	DB	Y	Co	F	0	1	V
*GM3594-70*	3	I	I	DB	Y	CC	F	0	2	V
*GM3650-51*	1	I	VS	DB	Y	Co	F	0	1	V
*Eco-Blanca*	4	I	I	DB	W	Co	F	0	2	V
*Llanera*	3	I	I	DB	W	Cy	F	0	2	V
*Cauca River Valley* (*CRV*) *Subregion*										
*SM3677-74*	2	L	I	LB	Y	Cy	F	0	1	V
*GM3594-70*	3	L	I	DB	Y	Co	F	0	3	V
*GM3650-51*	2	L	I	LB	Y	Co	F	0	2	V
*P13*	2	I	A	DB	W	Cy	F	0	4	I

Types: 1 = Excellent; 2 = Good; 3 = Average; 4 = Poor; 5 = Very poor. Lengths: A: Absent; VS = Very short; S = Short; I = Intermediate; L = Long; VL = Very long. Skin color: DB: Dark brown; LB: Light brown. Flesh color: Y: Yellow; W: White. Shapes: Co: Conical; CC: Conical/cylindrical; Cy: Cylindrical. Constrictions: A: Absent; F: Few; S: Several; M: Many. Vigor: TV: Too vigorous; V: Vigorous; I: Intermediate; W: Weak; TW: Too weak.

**Table 5:** Root quality results for key variables in cassava genotypes for fresh consumption, featuring low cyanogenic potential (HCN) and high β-carotene and total carotenoids contents, evaluated across four locations in two subregions. Values expressed on a fresh weight basis.

Genotype	Subregion	HCN (µg/g)	β-Carotene (µg/g)	Total Carotenoids (µg/g)
*GM3594-70*	Pacific	15.9	5.9	11.6
	Cauca River	9.6	7.9	13.0
*SM3677-74*	Pacific	49.3	5.9	10.4
	Cauca River	26.6	7.2	14.0
*GM3650-51*	Pacific	19.0	5.1	11.4
	Cauca River	17.8	2.7	8.8
*Llanera*	Pacific	28.1	0.4	1.2
*Eco-Blanca*	Pacific	16.1	0.7	1.1
*P13*	Cauca River	14.6	0.7	1.4

**Table 6:** Results of consumer acceptability and cooking tests for cassava genotypes intended for fresh consumption with low HCN and high carotenoid content, evaluated in four locations across two subregions.

Genotype	Subregion	Color	Taste	Texture	Average	Cooking Time (min)
*GM3594-70*	Pacifico	2.6	2.1	2.1	2.3	30
	Río cauca	3.9	3.9	4.0	3.9	25
*GM3650-51*	Pacifico	2.6	2.3	2.3	2.4	30
	Río cauca	3.9	4.0	4.1	4.0	10
*SM3677-74*	Pacifico	2.6	2.3	2.5	2.5	25
	Río cauca	3.9	3.0	3.1	3.3	30
*Llanera*	Pacifico	4.1	3.1	3.3	3.5	20
*Eco-Blanca*	Pacifico	3.1	3.3	3.3	3.3	15
*P13*	Río cauca	3.4	3.0	3.1	3.2	20

The description of color, flavor, and texture was based on a rating scale in which 1 indicated strong dislike for the evaluated attribute, 2 slight dislike, 3 indifference, 4 slight liking, and 5 liking.

## Data Availability

The original contributions presented in this study are included in the article. Further inquiries can be directed to the corresponding author.

## References

[1] Burns A., Gleadow R., Cliff J., Zacarias A., Cavagnaro T. (2010). Cassava: The drought, war and famine crop in a changing world. Sustainability.

[2] Caccamisi D.S. (2010). Cassava: Global production and market trends. Chronica Hort..

[3] FAO (2024). World Food and Agriculture—Statistical Yearbook.

[4] Agronet Evaluaciones Agropecuarias Municipales EVA Datos de 2024. https://www.agronet.gov.co/estadistica/Paginas/home.aspx?cod=1.

[5] FAO (2021). Cassava: A 21st Century Staple Crop. Food and Agriculture Organization of the United Nations. https://www.fao.org/4/i3278e/i3278e01.pdf.

[6] Howeler R., NeBambi L., Thomas G. (2013). Save and Grow: Cassava.

[7] Montagnac J.A., Davis C.R., Tanumihardjo S.A. (2009). Nutritional value of cassava for use as a staple food and recent advances for improvement. Compr. Rev. Food Sci. Food Saf..

[8] Bouis H.E., Saltzman A. (2017). Improving nutrition through biofortification: A review of evidence from HarvestPlus, 2003 through 2016. Glob. Food Sec..

[9] Talsma E.F., Brouwer I.D., Verhoef H., Mbera G.N., Mwangi A.M., Melse-Boonstra A. (2017). Biofortified yellow cassava and vitamin A status of Kenyan children: A randomized controlled trial. Am. J. Clin. Nutr..

[10] Ceccarelli S., Grando S., Maatougui M., Michael M. (2010). Plant breeding and climate changes. J. Agric. Sci..

[11] Ceballos H., Hershey C., Iglesias C., Zhang X. (2021). Fifty years of a public cassava breeding program: Evolution of breeding objectives, methods, and decision-making processes. Theor. Appl. Genet..

[12] Rodríguez E.P.B., Morante N., Salazar S., Hyde P.T., Setter T.L., Kulakow P., Zhang X. (2023). Flower-inducing technology facilitates speed breeding in cassava. Front. Plant Sci..

[13] Ma Q., Zhang P. (2025). Advancing cassava molecular breeding through genome editing: A promising pathway. Trop. Plants.

[14] Wolfe M.D., Del Carpio D.P., Alabi O., Egesi C., Rabbi I.Y. (2017). Prospects for genomic selection in cassava breeding. Plant Genome.

[15] Khoury C.K., Bjorkmoan A.D., Dempewolf H., Ramirez-Villegas J., Guarino L., Jarvis A., Rieseberg L.H., Struik P.C. (2014). Increasing homogeneity in global food supplies and the implications for food security. Proc. Natl. Acad. Sci. USA.

[16] Ilona P., Bouis H.E., Palenberg M., Oparinde A. (2017). Vitamin A Cassava in Nigeria: Crop Development and Delivery. Biofortification of Staple Crops.

[17] Rosero A., Ceballos H., León R., García J., Orozco A., Silva G., Montes M., Martínez R., Cordero C., de la Ossa V. (2025). Technical and Consumer Preferences Integrated for the Development of Cassava Varieties with High Nutritional Quality Adapted to Colombian Caribbean Coast. Plants.

[18] Saltzman A., Birol E., Oparinde A., Andersson M.S., Asare-Marfo D., Diressie M.T., Gonzalez C., Lividini K., Moursi M., Zeller M. (2017). Availability, production, and consumption of crops biofortified by plant breeding: Current evidence and future potential. Ann. N. Y. Acad. Sci..

[19] Ministerio de Salud y Protección Social, República de Colombia—MINSALUD (2011). Encuesta Nacional de la Situación Nutricional en Colombia (ENSIN 2010). https://www.icbf.gov.co/sites/default/files/resumenfi.pdf.

[20] Ministerio de Salud y Protección Social, República de Colombia—MINSALUD (2015). Estrategia Nacional para la Prevención y Control de las Deficiencias de Micronutrientes en Colombia 2014–2021. https://www.minsalud.gov.co/sites/rid/Lists/BibliotecaDigital/RIDE/VS/PP/SNA/Estrategia-nacional-prevencion-control-deficiencia-micronutrientes.pdf.

[21] Rosero A.E.A., Lascano H.C., Henao E.R. (2023). Aportes y Perspectivas del Mejoramiento Genético de Yuca en El Fortalecimiento de SU Red de Valor en Colombia.

[22] Gómez P., Losada J., Toro J. (1986). Maniohica P-13: Una Variedad Mejorada de Yuca de Alto Rendimiento [Boletín Divulgativo n.º 78].

[23] Valencia K.V.C., Alpala E.E.A.R. (2024). Comprensión Del Segmento de la Yuca de Consumo Fresco en Colombia. Manual de Manejo de Yuca Para Consumo Fresco en Diferentes Regiones de Colombia.

[24] Morillo Y., Sánchez T., Morante N., Chávez A.L., Morillo A.C., Bolaños A., Ceballos H. (2012). Estudio preliminar de herencia del contenido de carotenoides en raíces de poblaciones segregantes de yuca (*Manihot esculenta* Crantz). Acta Agronómica.

[25] Ceballos H., Morante N., Sánchez T., Ortiz D., Aragón I., Chávez A.L., Pizarro M., Calle F., Dufour D. (2013). Rapid Cycling Recurrent Selection for Increased Carotenoids Content in Cassava Roots. Crop Sci..

[26] León R.I., Rosero A., García J.-L., Morelo J., Orozco A., Silva G., De la Ossa V., Correa E., Cordero C., Villalba L. (2021). Multi-trait selection indices for identifying new cassava varieties adapted to the Caribbean region of Colombia. Agronomy.

[27] Essers S.A., Bosveld M., Van Der Grift R.M., Voragen A.G. (1993). Studies on the quantification of specific cyanogens in cassava products and introduction of a new chromogen. J. Sci. Food Agric..

[28] Martínez-Becerra R., Martínez-Rueda N., Martínez-Martínez V. (2011). Diseño de Experimentos en Ciencias Agropecuarias y Biológicas con SAS, SPSS, R y STATISTIX.

[29] Littell R.C., Milliken G.A., Stroup W.W., Wolfinger R.D., Oliver S. (2006). SAS for Mixed Models.

[30] Cuero L. (2010). Evaluación de Las Características Morfológicas Y Agronómicas de Tres Variedades de Yuca (*Manihot esculenta* Crantz), en Un Suelo Del Municipio de Buenaventura—Valle Del Cauca. https://repositorio.unipacifico.edu.co/entities/publication/91aa7f21-1af2-4a3f-bfe4-9fa5b20a2a67.

[31] Codex Alimentarius Commission (2013). Code of Practice for the Reduction of Hydrocyanic Acid (HCN) in Cassava and Cassava Products.

[32] Sánchez T., Alonso-A L., Ospina-P B., Ceballos H. (2002). Conservación Y Acondicionamiento de Las Raíces Frescas. La Yuca en el Tercer Milenio: Sistemas Modernos de Producción, Procesamiento, Utilización y Comercialización.

[33] Villwock S.S., Li L., Jannink J. (2024). Carotenoid-carbohydrate crosstalk: Evidence for genetic and physiological interactions in storage tissues across crop species. New Phytol..

[34] Sánchez T., Moreno R. (2016). Evaluación del contenido de materia seca y rendimiento en variedades de yuca para consumo y procesamiento. Rev. Fitotec. Mex..

[35] Ebdon J.S., Gauch H.G. (2002). Additive main effect and multiplicative interaction analysis of national turfgrass performance trials: I. Interpretation of genotype × environment interaction. Crop Sci..

[36] Ogwuche T.O., Diebiru-Ojo M.E., Najimu A., Ossai C.O., Ekanem U., Adegbite B., Oyebode G., Kulakow P. (2023). Performance and stability of improved cassava (*Manihot esculenta* Crantz) clones in demand creation trials in Nigeria. Crops.

[37] Oliveira E.J., de Oliveira G.C., Ratke C.L. (2018). Stability and adaptability of cassava genotypes based on combined analysis across environments. Euphytica.

[38] Cardoso A.P., Mirione E., Ernesto M., Massaza F., Cliff J., Haque M.R., Bradbury J.H. (2005). Processing of cassava roots to remove cyanogens. J. Food Compos. Anal..

[39] Srihawong W., Kongsil P., Petchpoung H., Sarobol E. (2015). Effect of Genotype, Age and Soil Moisture on Cyanogenic Glycosides Content and Root Yield in Cassava (*Manihot esculenta* Crantz). Agric. Nat. Resour..

[40] Esuma W., Kawuki R.S., Herselman L., Labuschagne M.T. (2016). Stability and genotype by environment interaction of provitamin A carotenoid and dry matter content in cassava in Uganda. Breed. Sci..

[41] Nduwumuremyi A., Melis R., Shanahan P., Theodore A. (2017). Interaction of genotype and environment effects on important traits of cassava (*Manihot esculenta* Crantz). Crop. J..

[42] Iragaba P., Adinsi L., Delgado L.F., Nanyonjo A.R., Nuwamanya E., Wembabazi E., Kanaabi M., Honfozo L., Hotegni F., Djibril-Moussa I. (2024). Definition of sensory and instrumental thresholds of acceptability for selection of cassava genotypes with improved boiling properties. J. Sci. Food Agric..

[43] Tang C., Heymann H. (2002). Multidimensional sorting, similarity scaling and free-choice profiling of grape jellies. J. Sens. Stud..

[44] Rosero A., Pastrana I., Martínez R., Perez J.-L., Espitia L., Araujo H., Belalcazar J., Granda L., Jaramillo A., Gallego-Castillo S. (2022). Nutritional value and consumer perception of biofortified sweet potato varieties. Ann. Agric. Sci..

